# Microstructure evolution with varied layer thickness in magnetron-sputtered Ni/C multilayer films

**DOI:** 10.1038/srep31522

**Published:** 2016-08-12

**Authors:** Jichang Peng, Wenbin Li, Qiushi Huang, Zhanshan Wang

**Affiliations:** 1 Key Laboratory of Advanced Micro-Structured Materials MOE, Institute of Precision Optical Engineering, School of Physics Science and Engineering, Tongji University, Shanghai 200092, China

## Abstract

The microstructure evolution of magnetron-sputtered Ni/C multilayers was investigated by varying the Ni and C layer thickness in the region of a few nanometers. For the samples having 2.6-nm-thick C layers, the interface width increases from 0.37 to 0.81 nm as the Ni layer thickness decreases from 4.3 to 1.3 nm. Especially for the samples with Ni layers less than 2.0 nm, the interface width changes significantly due to the discontinuously distributed Ni crystallites. For the samples having 2.8-nm-thick Ni layers, the interface width increases from 0.37 to 0.59 nm when the C layer thickness decreases from 4.3 to 0.7 nm. The evolution of interface microstructures with varied Ni and C layers is explained based on a proposed simple growth model of Ni and C layers.

Nanometer-scale multilayer films used as artificial Bragg reflectors are required for a variety of applications in the research fields, such as EUV photolithography, X-ray microscopy, synchrotron radiation, plasma physics, and astrophysics^1^,^2^,^3^. As an outstanding candidate, Ni/C material combination is quite often studied as multilayer mirrors working in EUV and X-ray energy region. Theoretically, Ni/C multilayers have high normal incidence reflectivities in the ‘carbon-window’ wavelength region^4^,^5^ due to a large electron density contrast. They are also suited as monochromators for Cu Kα radiation in virtue of low divergence of the reflected beam and the superior suppression of Cu Kβ radiation^6^,^7^,^8^. Moreover, depth-graded Ni/C multilayers are used in astronomical telescopes in hard X-ray region^9^,^10^.

For laterally- and depth-graded Ni/C multilayers, both Ni and C layer thicknesses are varied in certain ranges. For laterally-graded Ni/C multilayers used as “Göbel Mirrors” in X-ray diffractometry, the period thickness was designed in the range of 3–6 nm^11^. For depth-graded Ni/C multilayers applied in astronomical hard X-ray telescopes up to 100 keV, the periodic thickness was designed in the range of 2–30 nm^12^,^13^,^14^. When the periodic thickness decreases, the interface width including both contributions from interfacial roughness and interdiffusion may be accordingly changed, which has strong effects on the performances of multilayer mirrors, especially when the layers become thinner. For Ni/C multilayers, it has been observed that the reflectivity decreased dramatically when the periodic thickness is less than 4 nm, which is probably caused by the crystallization of Ni at a critical thickness of 2 nm and the interdiffusion between the adjacent layers of nickel and carbon^15^,^16^,^17^. The critical thickness for Ni crystallization has been found to be reduced by other deposition techniques such as PLD and IBSD^18^,^19^, since the deposited particles with higher kinetic energy can smooth the interfaces of Ni/C multilayers^20^. For magnetron-sputtered method, the kinetic energy of sputtered particles is typically less than 10 eV, while IBSD and PLD methods can generate sputtered particles with higher kinetic energy of up to 40 and several 100 eV, respectively. Nevertheless, it is still hard to deposit Ni/C multilayers with a period thickness below 3 nm. For applying laterally- and depth-graded Ni/C multilayers in hard X-ray region, it is highly required to investigate the evolution of interfacial microstructures with respect to the variation of Ni and C layer thickness, especially in a few nanometers layer thickness region.

In this paper, we present the investigations on microstructure evolutions with varied layer thickness for magnetron-sputtered Ni/C multilayers. Both effects of varied thicknesses of Ni and C layers on the interfacial microstructures were studied by characterization techniques including grazing incidence X-ray reflectivity (GIXRR), atomic force microscopy (AFM), high-resolution cross-sectional transmission electron microscopy (HRTEM), and selected area electron diffraction (SAED). A growth model based on the island growth of Ni on amorphous C is proposed to illustrate the effects of varied layer thickness on the interfacial microstructures of Ni/C multilayers.

## Results

### Grazing incidence X-ray reflectivity

**Figure 1:**
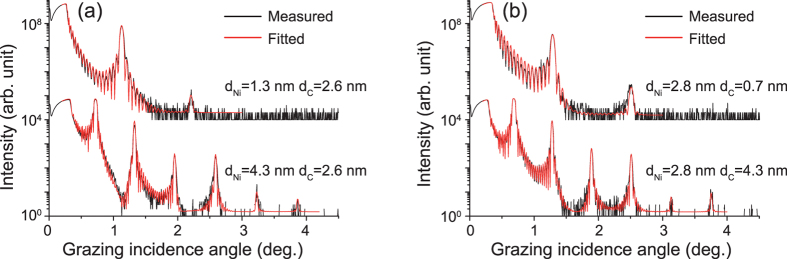
Measured and fitted GIXRR curves for the 1^st^ group Ni/C multilayers having 2.6-nm-thick C layers (**a**) and for the 2^nd^ group samples having 2.8-nm-thick Ni layers (**b**).

GIXRR measurements were performed on all samples and two typical experimental data for each group with the thinnest and the thickest Ni or C layer are selected and presented in Fig. 1. Shown in Fig. 1a are the measured GIXRR curves for the 1^st^ group of samples with the fixed carbon layer thickness of 2.6 nm. Only two Bragg peaks can be observed for the sample with the thinnest Ni layers (d_Ni_ = 1.3 nm), compared with six Bragg peaks observed for the other sample with the thickest Ni layers (d_Ni_ = 4.3 nm). This indicates that there is a larger interface width for the sample with thinner Ni layers. The GIXRR data for the 2^nd^ group of samples with the fixed nickel layer thickness of 2.8 nm are displayed in Fig. 1b. Similarly, it is found that there are less Bragg peaks for the sample with the thinnest C layers (d_C_ = 0.7 nm) compared with the sample with the thickest C layers (d_C_ = 4.3 nm).

**Figure 2:**
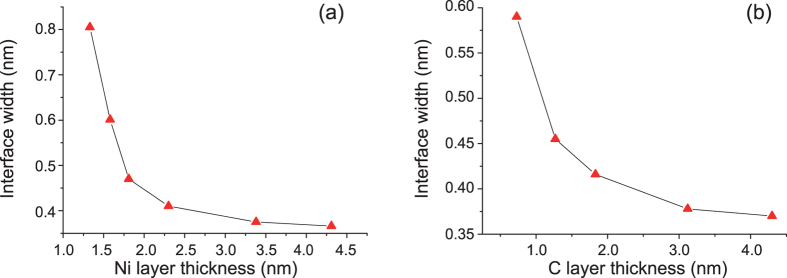
The average interface widths determined by fitting the GIXRR data as a function of varied layer thickness for Ni/C multilayers having 2.6-nm-thick C layers (**a**), and for samples having 2.8-nm-thick Ni layers (**b**).

By utilizing Bede Refs software^21^, the measured GIXRR data were fitted to determine the average layer thickness and interface width. As shown in Fig. 1, the fitting curves agree quite well with the corresponding experimental data. The fitted average interface widths are given in Fig. 2a as a function of Ni layer thickness for the 1^st^ group samples with 2.6-nm-thick C layers. It can be seen that the interface width increases from 0.37 nm to 0.81 nm as the thickness of Ni layer decreases from 4.3 nm to 1.3 nm. In addition, there is a critical thickness at about 2.0 nm for Ni layer, below which the interface width increases rapidly from about 0.45 nm to 0.81 nm. This critical thickness of Ni layers is consistent with the results reported by Borchers *et al*.^16^, where the reflectivity of Ni/C multilayer dropped rapidly at a periodic thickness of 4.0 nm with d_Ni_ = 2.0 nm. For the 2^nd^ group samples with 2.8-nm-thick Ni layer, the fitted average interface widths are given in Fig. 2b as a function of C layer thickness. The interface width increases approximately from 0.37 nm to 0.59 nm when the thickness of C layers decreases from 4.3 nm to 0.7 nm. Comparing these two curves as shown in Fig. 2, it is found that the increase of interface widths for samples with thinner C layers are less than that with thinner Ni layers in the thickness region less than 2.0 nm. So, it can be deduced that the deposited carbon atoms can form continuous films in thinner layers than Ni layers.

### Atomic force microscopy

**Figure 3:**
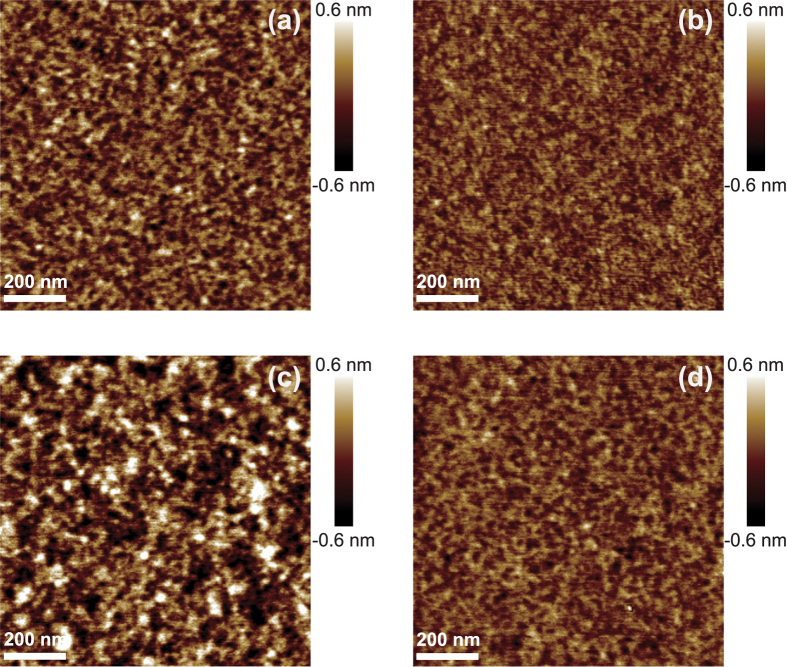
AFM images of selected Ni/C multilayers with different Ni and C layer thickness: (**a**) d_Ni_ = 1.3 nm, d_C_ = 2.6 nm, (**b**) d_Ni_ = 4.3 nm, d_C_ = 2.6 nm, (**c**) d_Ni_ = 2.8 nm, d_C_ = 0.7 nm, and (**d**) d_Ni_ = 2.8 nm, d_C_ = 4.3 nm.

Shown in Fig. 3a,b are the AFM measurement results for the selected samples with 2.6-nm-thick C layers: (a) d_Ni_ = 1.3 nm and (b) d_Ni_ = 4.3 nm. For each sample, three different positions have been measured to determine the surface roughness (root mean square, RMS). It is found that the surface roughness increases just slightly from 0.13 nm to 0.17 nm as the Ni layer thickness decreases from 4.3 nm to 1.3 nm. Since the surface roughness is significantly smaller than the interface width determined by GIXRR, it can be inferred that the interdiffusion between Ni and C layers contributes partly to the interfacial width. Nevertheless, as revealed in next section, the interfaces become much rougher when the Ni layer thickness decreases from 4.3 nm to 1.3 nm because of the formation of Ni grains. Therefore, a rougher surface is expected for the sample with d_Ni_ = 1.3 nm if the spatial distribution of Ni grains is replicated as a height modulation on the top surface C layer. However, it is not the case for the sample shown in Fig. 3a compared with the other sample shown in Fig. 3b. So, the small surface roughness may be contributed by the smoothing effects of the C layer with a thickness of 2.6 nm on the top^22^,^23^,^24^. Figure 3c,d give the AFM measurement results for the selected samples with 2.8-nm-thick Ni layers: (c) d_C_ = 0.7 nm, and (d) d_C_ = 4.3 nm. In contrary to the results shown in Fig. 3a,b, the surface roughness increases from 0.13 nm to 0.27 nm as the C layer thickness decreases from 4.3 nm to 0.7 nm. For this case, the C layer may not grow continuously anymore when it becomes extremely thin, for example 0.7 nm, which causes a rough surface.

### High resolution transmission electron microscopy

**Figure 4:**
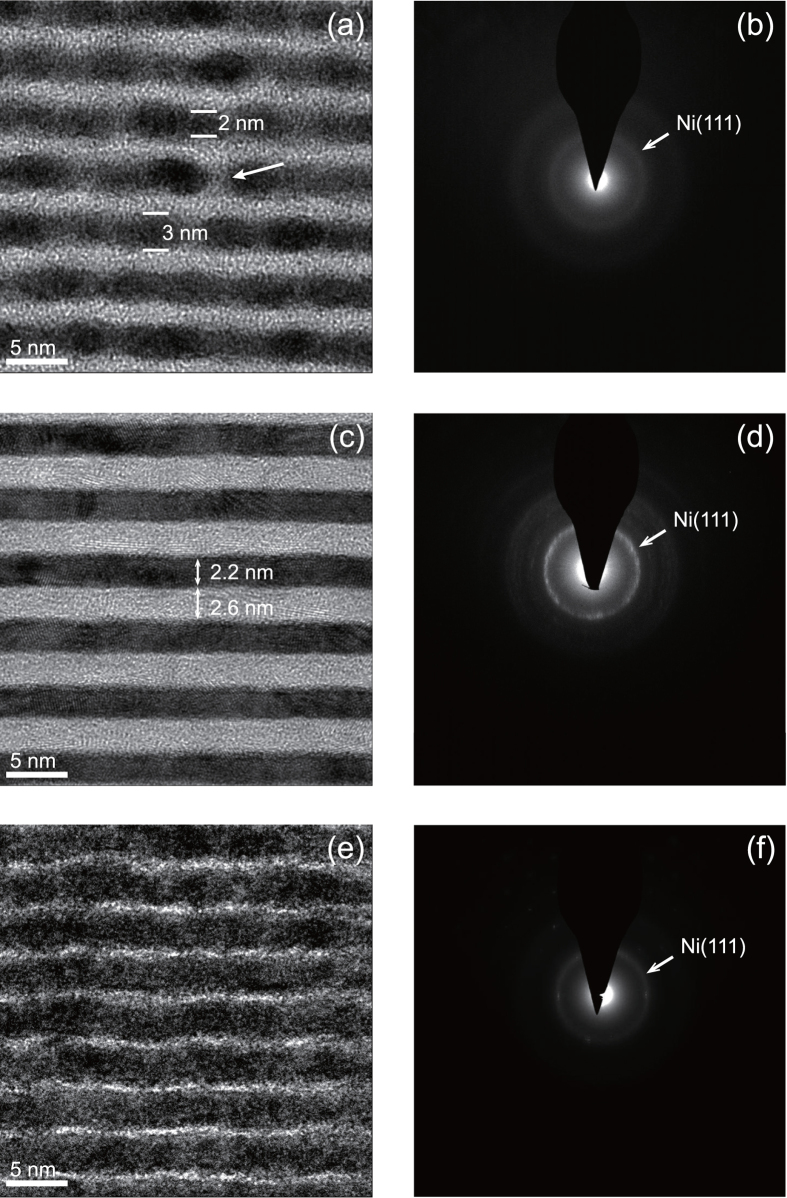
High resolution TEM images and the electron diffraction patterns of selected Ni/C multilayers: (**a,b**) d_Ni_ = 1.3 nm d_C_ = 2.6 nm, (**c,d**) d_Ni_ = 2.2 nm d_C_ = 2.6 nm, and (**e,f**) d_Ni_ = 2.8 nm d_C_ = 0.7 nm.

High resolution TEM images for selected Ni/C multilayers are shown in Fig. 4, where the dark layers are Ni and the bright layers are C. For the sample having the thinnest Ni layer (d_Ni_ = 1.3 nm, d_C_ = 2.6 nm), a periodic layered structure can still be clearly observed in Fig. 4a, but with quite rough interfaces between the adjacent layers. Some grains with ellipsoidal shapes are randomly distributed inside the Ni layers. The sizes of these Ni grains along the multilayer growth direction are about 2–3 nm, which is generally smaller than the lateral sizes of these grains but larger than the average Ni layer thickness determined by GIXRR. So, the formation of relatively larger Ni grains than the average layer thickness increases the interface roughness. Additionally, since these grains are isolated from each other or just partially in contact, there are some gaps or interstices between them, which are filled up with the amorphous carbon as indicated in Fig. 4a by an arrow. Therefore, the interdiffusion between the adjacent Ni and C layers becomes larger when the Ni layers are thinner than the grain size at about 2.0 nm. Moreover, the filling process by the amorphous carbon may induce some smoothing effects when deposited on Ni layers by damping the high frequency components of the interface roughness^23^. The SAED image from this specimen is shown in Fig. 4b. One diffraction ring was observed corresponding to the Ni(111) planes diffraction with Ni fcc structure. The observed phase is consistent with the reported XRD results^25^,^26^. No obvious intensity modulations are observed in this blurred diffraction ring, which indicates that these Ni crystalline grains have no preferred orientation inside the Ni layers. From the results given in Fig. 4a,b, it can be concluded that the rough interfaces of Ni/C multilayer with the thinnest Ni layers (d_Ni_ = 1.3 nm) are mainly caused by these polycrystalline Ni grains distributed randomly in the Ni layers. This fact has also been found by Borchers *et al*. in the magnetron sputtered Ni/C graded multilayers^16^.

The high resolution TEM image for the sample with intermediate thicknesses of Ni (d_Ni_ = 2.2 nm, d_C_ = 2.6 nm) is shown in Fig. 4c. It can be seen that the interfaces between Ni and C layers become quite smooth and sharp, which is consistent with the interface width determined by GIXRR. As seen in Fig. 4c, the Ni layers are mostly polycrystalline identified by the lattice fringes with different orientations. This fact is in agreement with the SAED image as shown in Fig. 4d, where an intense diffraction ring is presented corresponding to fcc Ni(111) planes. These crystalline grains without preferred orientations are distributed in almost the whole Ni layers, where no obvious interstices between grains can be identified as for the case shown in Fig. 4a. The carbon layers are still amorphous since no other diffraction rings from carbon can be seen. By comparing the different microstructures shown in Fig. 4a,c, it can be deduced that the smooth interfaces of the sample with Ni layers thickness of 2.2 nm are mainly contributed by the crystallization occurring almost in the whole Ni layers. To study the effects of thin C layers on interface microstructures, the high resolution TEM and the SAED images for the sample having the thinnest C layer (d_Ni_ = 2.8 nm, d_C_ = 0.7 nm) are given in Fig. 4e,f, respectively. The location and relative intensity distribution of the diffraction ring are similar to that of the sample displayed in Fig. 4d, which indicates that the Ni layers are also in a polycrystalline state since both samples have the similar thickness of Ni layers. Comparing Fig. 4e with Fig. 4c, it can be seen that the interfaces are quite rough when the C layers are just 0.7 nm, which is consistent with the results determined by GIXRR and AFM for the same sample. The only difference between the samples as shown in Fig. 4c,e is the thickness of C layers. Therefore, it can be inferred that the thin C layers with 0.7 nm grow discontinuously and causes the rough interfaces, which is also evidenced by the AFM measurement as shown in Fig. 3c.

## Discussion

**Figure 5:**
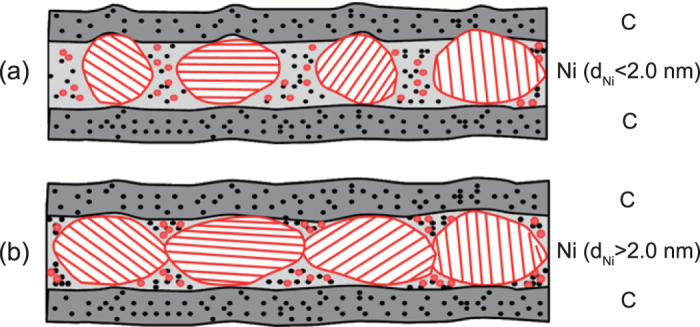
A growth model for the interface microstructures of Ni/C multilayers for the case of (**a**) d_Ni_ < 2.0 nm and (**b**) d_Ni_ > 2.0 nm.

Our experimental results demonstrate that the interface microstructures of Ni/C multilayer are correlated with the crystallization of Ni layers, the smoothing effect of thick C layers and discontinuous growth of thin C layers. To illustrate the effects of varied layer thickness on the interface microstructures, a growth model is proposed as schematically shown in Fig. 5. During the growth of Ni on an amorphous C layer, the Ni layer begins with island formations induced by the nonwetting condition^16^. No obvious formation of Ni-on-C or C-on-Ni interlayers was found in the TEM images, which is consistent with the prediction of a limited solid solubility of C in Ni and zero solubility of Ni in C in the Ni-C phase diagram^27^ though these Ni/C multilayers are formed in a non-equilibrium process. Based on the experimental results, the critical thickness for the formation of Ni crystallites is about 2.0 nm. As shown in Fig. 5a, even when the average thickness of Ni layer is less than the critical thickness, the Ni layer can still sustain Ni crystallites with the size of about 2–3 nm along the growth direction. Additionally, these polycrystalline Ni grains have different orientations inside the layer as presented in Fig. 5. Because of the randomly distributed grains, the Ni layer is discontinuous with some gaps and interstices between them and the interface is quite rough as the case shown in Fig. 4a. A similar phenomenon has been observed on Mo/Si multilayer by Bajt *et al*.^28^. The subsequently deposited thick carbon layer will fill up these gaps and interstices and form an amorphous layer. Therefore, the interface widths of Ni/C multilayers with Ni layers thinner than 2.0 nm are quite large because of the large interface roughness and the enhanced interdiffusion. The thick C layer may have some smoothing effects by damping the high frequency components of the interface roughness caused by the formation of Ni grains^23^, which is evidenced by the similar top surface roughness for the samples with Ni layer thickness of 1.3 and 4.3 nm (Fig. 3a,b).

As shown in Fig. 5b, when the thickness of Ni layer is above the critical thickness of 2.0 nm, the coalescence of these Ni grains occurs and thus the Ni layer becomes continuous with a less rough interface. Consequently, smooth and sharp interfaces are presented for the sample as shown in Fig. 4c. When the C layer is quit thin, for example 0.7 nm as the case in Fig. 4e, the C layer cannot grow continuously anymore. For these reason, as revealed by AFM, the surface roughness becomes rougher for the samples when the C layer thickness decreases from 4.3 nm to 0.7 nm (Fig. 3c,d).

In conclusion, the effects of both Ni and C layer thickness on the interface microstructures of Ni/C multilayers were investigated by a variety of characterization techniques. It is found that the interface width is strongly dependent on the crystallization of Ni layers, the smoothing effects of thick C layers and the discontinuous growth of thin C layers. For the samples with 2.6-nm-thick C layers, where the smoothing effects of C layers are almost identical, the interface width decreases significantly when the thickness of Ni layers increases across the critical thickness at about 2.0 nm due to the formation of Ni crystallites. For the samples with 2.8-nm-thick Ni layers, where the Ni layers are mostly crystallized, the interface width increase rapidly up to 0.59 nm when the C layers are just 0.7 nm, which can be caused by the discontinuous growth and insufficient smoothing effects of C layers on the underlying Ni layers. The revolution of the interface width is explained by a simple model based on the growth of Ni and C layers. The present investigations provide some useful clues for fabricating graded Ni/C multilayer where both Ni and C layers thickness are varied in the region of a few nanometers. For preparing an optimized depth-graded Ni/C multilayer, the formation of discontinuously distributed Ni crystallites and the discontinuous growth of C layers should be avoided. Therefore, the minimum thickness of Ni layers and C layers should be larger than 2.0 nm and 1.4 nm, respectively, in order to obtain Ni/C multilayer with interface width less than 0.45 nm.

## Methods

The Ni/C multilayers were deposited on super-polished silicon wafers with size of 20 mm × 20 mm using direct-current (DC) magnetron sputtering at room temperature. The base pressure was 2.7 × 10^−4^ Pa and the pressure of Argon sputtering gas with purity of 99.999% was maintained at 1.5 mTorr during deposition. The powers and voltages applied on Ni and C targets were held constantly at 20 W/320 V and 100 W/460 V, respectively. For all Ni/C multilayer samples, each of them contains 20 bilayers with the bottom layer of Ni and the top layer of C. All samples are divided into two groups. For the first group, the thickness of Ni layers varied from 1.3 to 4.3 nm with the C layers kept at about 2.6 nm. For the second group, the thickness of C layers varied from 0.7 to 4.3 nm with the Ni layers kept at about 2.8 nm. The individual layer thickness of Ni or C was controlled by the relevant deposition time.

The layer structures of the samples were first characterized by GIXRR measurements based on Bede D1 X-ray diffractometer using a Cu Kα source (λ = 0.154 nm). The surface topographies of selected samples were measured using an atomic force microscope (Bruker DI D3100). Furthermore, high resolution transmission electron microscopy and selected area electron diffraction, provided by Materials Analysis Technology Inc., were used to investigate the layer morphology and crystalline structure. The TEM samples were prepared by focused ion beam milling and measured using a FEI Tecnai G2 F20 instrument operating at 200 keV.

## Additional Information

**How to cite this article**: Peng, J. *et al*. Microstructure evolution with varied layer thickness in magnetron-sputtered Ni/C multilayer films. *Sci. Rep.* **6**, 31522; doi: 10.1038/srep31522 (2016).
